# LINC00511 promotes proliferation and invasion by sponging miR-515-5p in gastric cancer

**DOI:** 10.1186/s11658-020-0201-x

**Published:** 2020-02-03

**Authors:** Di Wang, Ketong Liu, Erdong Chen

**Affiliations:** 1 Department of Gastroenterology I, The Third Affiliated Hospital of Qiqihar Medical University, Qiqihar, 161000 Heilongjiang China; 2 Department of Cardiology III, The Third Affiliated Hospital of Qiqihar Medical University, Qiqihar, 161000 Heilongjiang China

**Keywords:** Gastric cancer, LINC00511, miR-515-5p, ceRNA

## Abstract

**Background:**

Long non-coding RNAs (lncRNAs) are known to be involved in tumorigenesis. The functions of LINC00511 in gastric cancer are poorly understood.

**Methods:**

Quantitative RT-PCR was performed to investigate the levels of LINC00511 in gastric cancer tissues and cell lines. CCK-8, flow cytometry, wound-healing and Transwell assays were performed to examine cellular functions. The underlying regulatory mechanisms of LINC00511 in gastric cancer progression were determined using luciferase reporter and RIP assays.

**Results:**

LINC00511 levels were significantly higher in gastric cancer tissues and cell lines than in normal samples. The high expression of LINC00511 in gastric cancer patient samples positively correlated with advanced clinical characters and poor prognosis. Depleting LINC00511 reduced tumor cell proliferation, migration and invasion, slowed tumor growth, and accelerated cell apoptosis. Our mechanistic study results indicated that LINC00511 promotes gastric cancer progression in a miR-515-5p-dependent manner.

**Conclusion:**

We established that LINC00511 may contribute to the proliferation and invasion of gastric cancer cells by modulating miR-515-5p, indicating that LINC00511 may be a potential molecular target for the development of anti-cancer drugs.

## Introduction

Gastric cancer is the fifth most common cancer and the third major cause of cancer-related mortality worldwide [1, 2]. Current treatment approaches consist of a combination of surgery, radiation and chemotherapy. Although treatment advancements have been made, the prognosis remains unsatisfactory due to metastasis and chemoresistance [3, 4]. It is vital to understand the molecular mechanisms for the growth and metastasis of gastric cancer and identify effective treatment approaches.

Long non-coding RNAs (lncRNAs) are RNA molecules that are longer than 200 nucleotides and incapable of being translated into proteins [5]. Various reports have shown that lncRNAs participate in growth, metastasis, differentiation and apoptosis, among other processes [6, 7]. Zhou et al. showed that a decrease in the levels of the lncRNA LET correlated with a worse prognosis in gastric cancer patients [8]. Li et al. suggested that CASC2 suppressed gastric cancer cell proliferation through the MAPK pathway [9]. Liu et al. revealed that GAS5 acted as a molecular sponge, regulating miR-23a expression in gastric cancer [10].

We explored the function of the lncRNA LINC00511 on the development and metastasis of gastric cancer and the underlying mechanisms. Our results reveal for the first time that LINC00511 promotes the growth and invasion of gastric cancer cells through sponging miR-515-5p. This discovery provides a possible new treatment target for this malignant tumor.

## Materials and methods

### Patients and specimens

The subjects were 25 patients who had been diagnosed with gastric cancer (age range, 36–81; 14 male patients and 11 female patients) and were due to undergo surgery in The Third Affiliated Hospital of Qiqihar Medical University. None of the patients received radiotherapy or chemotherapy prior to the operation. During the operation, tumor and corresponding healthy gastric tissue samples were collected and immediately snap frozen… The experimental protocols were approved by the Research Ethics Committee of The Third Affiliated Hospital of Qiqihar Medical University.

### Cell culture and transfection

A human gastric epithelial cell line (GES-1) and five human gastric cancer cell lines (AGS, SGC7901, BGC823, MKN45, MGC803) were obtained from then American Type Culture Collection (ATCC). All cells were grown in Dulbecco’s modified Eagle’s medium (DMEM; Invitrogen), which was augmented with 10% fetal bovine serum (FBS; Invitrogen), and cultured at 37 °C in a humidified atmosphere containing 5% CO_2_.

Short hairpin RNA targeting LINC00511 (sh-LINC00511) [11], miR-515-5p mimics, miR-515-5p inhibitors and scramble controls were customized by GenePharma and applied to co-transfected target cells per the manufacturer’s instructions.

### RNA extraction and quantitative RT-PCR analyses

TRIzol (Invitrogen) reagent was used to isolate 1 μg of total RNA and then reverse-transcribed into cDNA using PrimeScript RT Reagent Kit (Takara) according to the manufacturer’s instructions. Next, the SYBR Premix Ex Taq II kit (Takara) and the ABI 7500HT Real-Time PCR System (Applied Biosystems) were used to perform real-time PCR. Expression of RNA was normalized to either U6 or GAPDH and calculated using the CT method (2^-△△CT^). The primer sequences were: LINC00511, forward 5′-CTAACAAGAGGGTAAGTGTCAG-3′ and reverse 5′-AAGTCGACAACCCCATCGTTAC-3′ [11]; miR-515-5p, forward 5′-TTCTCCAAAAGAAAGCACTTTCTG-3′ and reverse 5′-CTCGCTTCGGCAGCACA-3′ [12]; GAPDH, forward 5′-CATCAAGAAGGTGGTGAAGCAG-3′ and reverse 5′-CGTCAAAGGTGGAGGAGTGG-3′; and U6, forward 5′-GCGCGTCGTGAAGCGTTC-3′ and reverse 5′-GTGCAGGGTCCGAGG-3′.

### GEPIA and Kaplan-Meier plotter databases

GEPIA data was used to obtain LINC00511 expression levels in stomach adenocarcinoma tissues and healthy tissues. The prognosis of gastric cancer patients was explored using the online Kaplan-Meier Plotter database.

### CCK-8 assay

After transfection, the cells were seeded onto 96-well plates (2 × 10^3^ cells/well) as indicated for 24, 48, 72 and 96 h. Next, 10 μl of the solutions from the Cell Counting Kit-8 (CCK-8; Roche) were added to every well and incubated for 2 h. Lastly, the optical density (OD) value of each well was quantified at 450 nm wavelength using a microplate reader (Bio-Rad).

### Colony formation assay

The colony formation assay was performed using the method detailed in a previous report [12].

### Flow cytometry assays

Cell apoptosis was determined using a BD Biosciences cell apoptosis kit per the manufacturer’s instructions. FACS flow cytometry (Beckman) was used to analyze apoptosis. It was also applied for the cell-cycle analysis, using the method detailed in a previous report [10].

### Wound-healing assay

Transfected cells (1 × 10^5^ cells/well) were seeded and plated onto 6-well plates until they reached confluence. Next, a homogeneous wound was generated in the monolayer using a sterile plastic micropipette tip. The wound closure was imaged after 0 and 24 h using an Olympus microscope.

### Transwell invasion assay

The top inserts of the Transwell chambers were coated with 50 μl of Matrigel (BD Biosciences) and left overnight at 4 °C. Then, 200 μl of cell suspension that contained 1 × 10^5^ transfected cells in serum-free medium was placed into the top inserts. After 48 h, the cells were fixed and stained with 0.1% crystal violet. The quantity of cells that had passed through the Matrigel were measured using an Olympus microscope.

### In vivo tumorigenesis

Animal experiments were approved by the Animal Care and Use Committee at Qiqihar Medical University. BALB/c nude mice that were 4–6 weeks old and weighed 18–20 g were obtained from the Laboratory Animal Research Centre at Qiqihar Medical University. 5 × 10^6^ cells transfected with either sh-LINC00511 or sh-NC were subcutaneously inoculated in the mice’s right flanks. The width (W) and length (L) of the xenografts that formed were quantified every 3 days. The tumor volume was estimated based on the formula

$$ \mathrm{Volume}\ \left(\mathrm{V}\right)=0.5\times \mathrm{L}\times {\mathrm{W}}^2 $$

After 21 days, the mice were killed via cervical dislocation and the xenografts were removed and weighed.

### Western blotting

Western blotting was conducted as described previously [9]. The primary antibodies included: ERK1/2 (cat. no. ab205718; 1:1000; Abcam), JNK (cat. no. ab131499; 1:1000; Abcam), p38 MAPK (cat. no. ab47363; 1:1000; Abcam), and GAPDH (1:10000; cat. no. sc-32,233; Santa Cruz Biotechnology). HRP-linked secondary antibodies were also brought from Santa Cruz Biotechnology. The levels of protein were revealed using ECL reagents (Amersham Biosciences).

### Immunohistochemical staining

Paraffin-embedded tissue sections were heated at 60 °C for 2 h, dewaxed with xylene and hydrated sequentially with gradient ethanol. The activity of endogenous peroxidase was inhibited using 3% hydrogen peroxide, and non-specific sites were blocked using serum. After antigen retrieval, the tissue sections were incubated with Ki-67 antibody (1: 500; Abcam) overnight at 4 °C. After washing, the tissue sections were incubated with homologous secondary antibody for 2 h at room temperature. Finally, the tissue sections were stained with a 3, 3′-diaminobenzidine solution and hematoxylin.

### Luciferase reporter assay

BGC823 cells (3 × 10^4^ cells/well) were planted into 24-well plates for 24 h and co-transfected with LINC00511-WT or LINC00511-Mut with or without miR-515-5p mimics using Lipofectamine 2000 reagents (Invitrogen). After 24 h, the cells were collected and luciferase activity was quantified using the Dual-Luciferase Reporter Assay System (Promega) according to the manufacturer’s instructions. Firefly luciferase activity was divided by Renilla luciferase activity for every sample to determine the transfection efficiency, which was used to normalize the data.

### RIP assay

An RNA immunoprecipitation (RIP) assay was performed as described previously [13].

### Statistical analysis

SPSS 16.0 software was used to conduct the statistical analysis. The results were evaluated as the means ± SD of at least 3 independent experiments. Differences between two groups were compared through an independent t-test. Multiple groups were compared using one-way ANOVA. *p* < 0.05 indicated statistical significance.

## Results

### LINC00511 was upregulated in gastric cancer

**Fig. 1:**
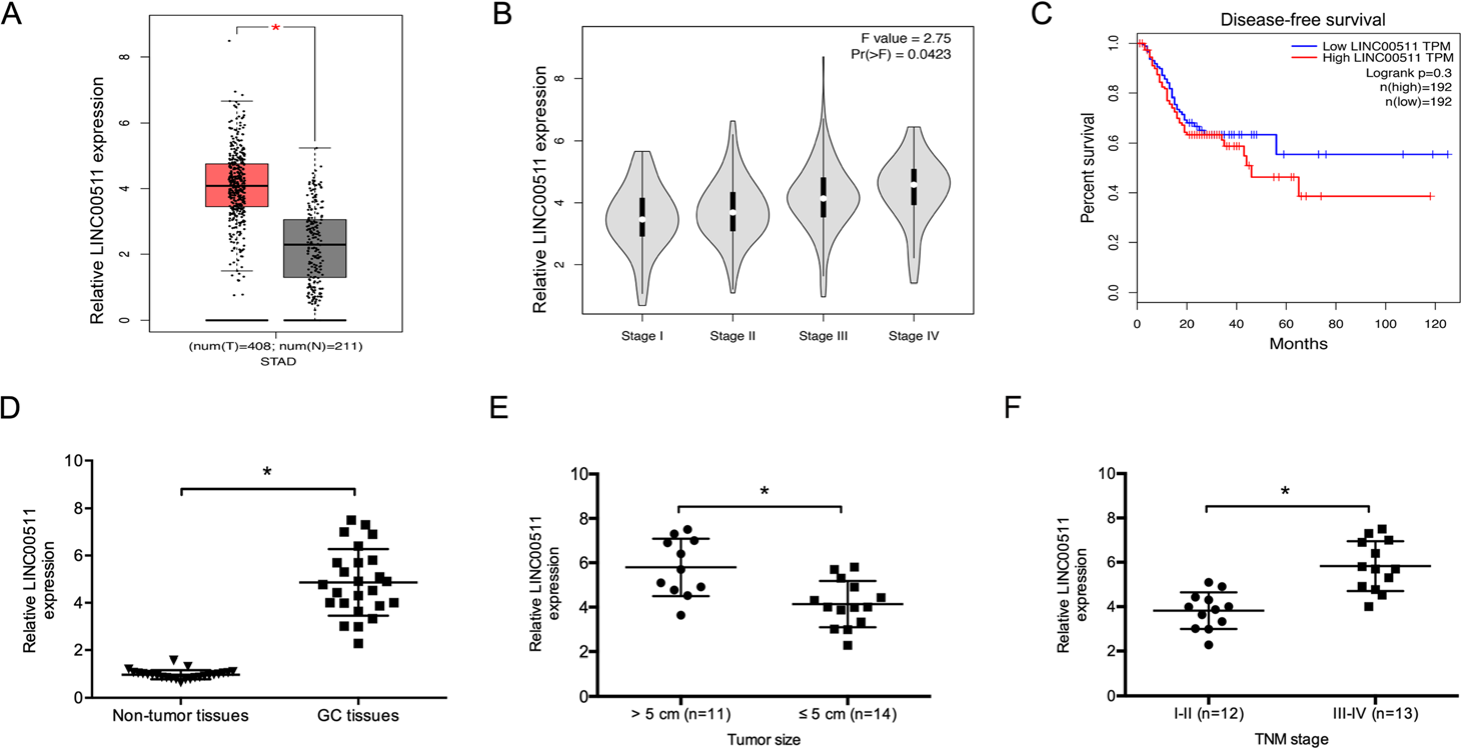
The LncRNA LINC00511 is upregulated in gastric cancer. **a** and **b** The relative LINC00511 expression in gastric cancer tissues, based on the TCGA dataset. **c** Disease-free survival analysis based on the TCGA dataset. **d** LINC00511 expression in 25 paired gastric cancer tissues was evaluated using quantitative RT-PCR. **e** and **f** High levels of LINC00511correlated with larger tumor size and advanced TNM stage in GC patients. *p < 0.05. Individuals were separated into two groups based on median gene expression

LINC00511 expression was determined to examine its function in gastric cancer progression. The GEPIA database revealed that LINC00511 is upregulated in stomach adenocarcinoma tissues and correlates with advanced tumor stage (Fig. 1a and b). Kaplan-Meier analysis showed that high LINC00511 levels predicted poor disease-free survival in gastric cancer patients (Fig. 1c).

We also evaluated LINC00511 expression in 25 paired gastric cancer tissues. LINC00511 expression was obviously higher in gastric cancer tissues (Fig. 1d). High LINC00511 expression levels correlated with bigger tumor size and advanced TNM stage in gastric cancer patients (Fig. 1e and f). Those results indicate that LINC00511 is involved in gastric cancer tumorigenesis.

### Knockdown of LINC00511 inhibited gastric cancer progression

**Fig. 2:**
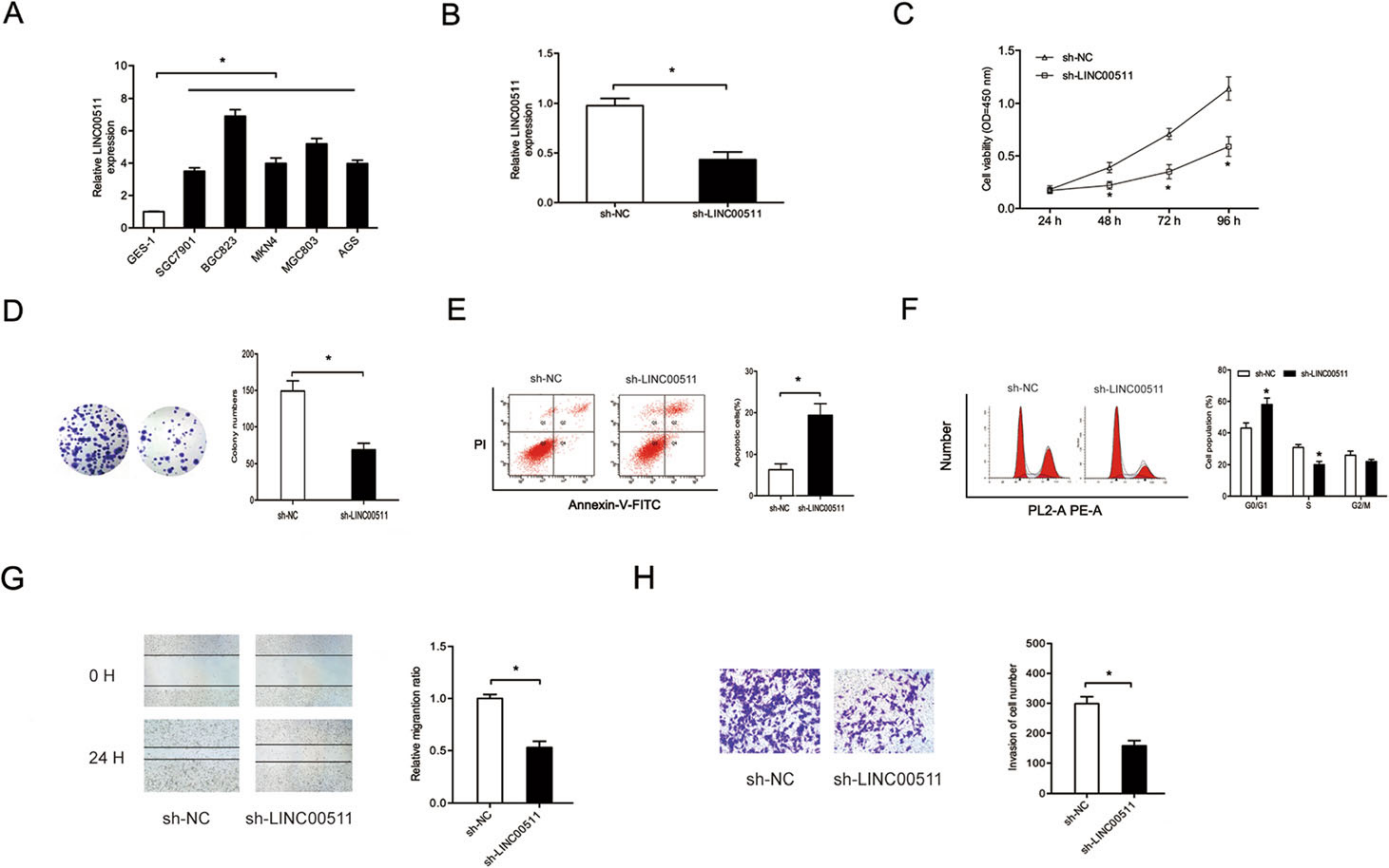
Knockdown of LINC00511 suppresses gastric cancer progression. **a** LINC00511 levels were evaluated in gastric cancer cell lines using quantitative RT-PCR. **b** The efficiency of knockdown of sh-LINC00511 in BGC823 cells was identified using quantitative RT-PCR. **c** and **d** Cell viabilities in BGC823 cells transfected with sh-LINC00511 were explored using the CCK-8 assay and colony formation assay (magnification, × 100). **e** and **f** Cell apoptosis and cell cycle distributions in BGC823 cells transfected with sh-LINC00511 were determined using flow cytometry assays. **g** and **h** LINC00511 inhibition significantly reduced BGC823 cell migration and invasion (magnification, × 200) ability. *p < 0.05

The functions of LINC00511 in gastric cancer progression were explored. Quantitative RT-PCR revealed that levels of LINC00511 were significantly higher in cells of the gastric cancer lines AGS, SGC7901, BGC823, MKN45 and MGC803 than in those of the human gastric epithelial cell line GES-1 (Fig. 2a). After sh-LINC00511 and sh-NC vectors were transfected into BGC823 cells (Fig. 2b), the CCK-8 assay showed LINC00511 knockdown significantly blocked their proliferative and colony formation ability (Fig. 2c and d). The flow cytometry assay revealed that LINC00511 knockdown increased apoptosis in BGC823 cells and arrested them in G0/G1 phase (Fig. 2e and f). Furthermore, the Transwell assay showed that LINC00511 inhibition decreased the invasive ability of BGC823 cells (Fig. 2g and h).

### Knockdown of LINC00511 reduced the activation of the MAPK signaling pathway

**Fig. 3:**
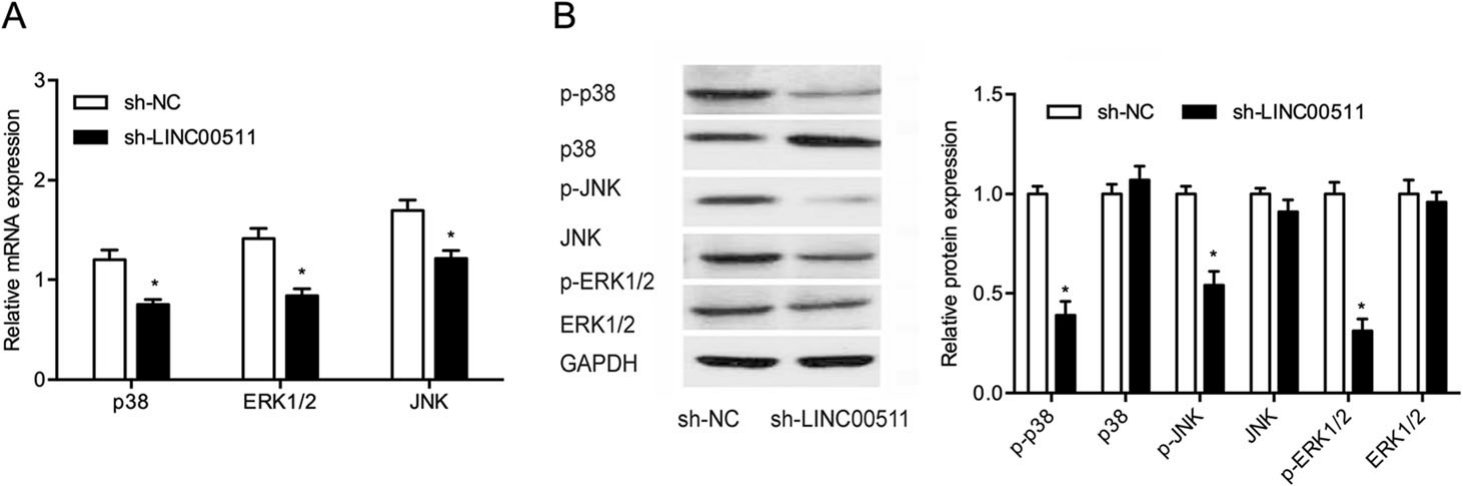
LINC00511 promotes the activation of one of the MAPK signaling pathways. **a** MAPK-related gene mRNA expression following LINC00511 in BGC823 cells. **b** The effects of LINC00511 knockdown on MAPK-related gene protein expression in BGC823 cells. *p < 0.05

The MAPK signaling pathway involving JNK, ERK and p38-MAPK, which play critical roles in tumor progression [14]. We explored the impact of LINC00511 on MAPK-related gene expression in BGC823 cells. Quantitative RT-PCR showed p38, ERK1/2 and JNK RNA levels were significantly lower in BGC823 cells transfected with sh-LINC00511 than in the sh-NC group (Fig. 3a). This is consistent with the protein expression levels of these genes (Fig. 3b). These findings support an LINC00511 contribution to gastric cancer progression through the MAPK signaling pathway.

### MiR-515-5p is a target of LINC00511

**Fig. 4:**
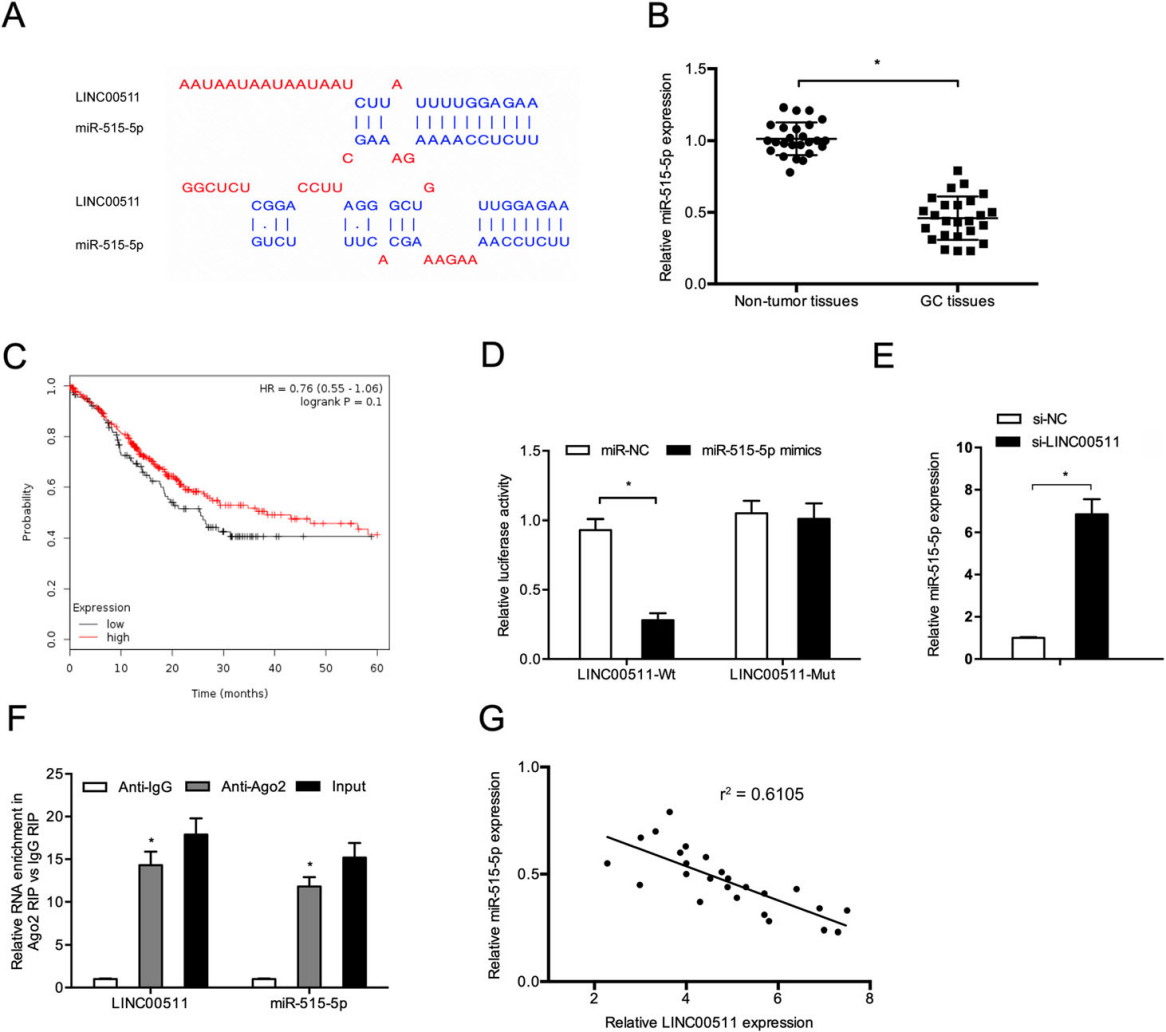
MiR-515-5p is a target of LINC00511 in gastric cancer. **a** The recognized binding sites between LINC00511 and miR-515-5p. **b** MiR-515-5p expression in 25 pairs of gastric cancer tissues (GC tissues) was evaluated using quantitative RT-PCR. **c** Low miR-515-5p levels correlated with poor survival in gastric cancer patients based on the TCGA dataset. **d** MiR-515-5p overexpression led to a reduction in luciferase activity in the LINC00511-Wt group. **e** LINC00511 knockdown increased miR-515-5p expression in BGC823 cells. **f** The RIP assay revealed that both LINC00511 and miR-515-5p expression are enhanced in the mixture immunoprecipitated by anti-Ago2. **g** LINC00511 expression negatively correlated with miR-515-5p expression in gastric cancer tissues. *p < 0.05

A previous report stated that lncRNAs could serve as a sponge for modulating the expression and activity of miRNA [15]. The target prediction tool DIANA was used to assess putative miRNAs that interact with LINC00511. It demonstrated that LINC00511 possesses a putative miR-515-5p-binding site (Fig. 4a). Quantitative RT-PCR data demonstrated that miR-515-5p levels were significantly lower and associated with poor overall survival in gastric cancer patients (Fig. 4c).

Next, we showed that miR-515-5p overexpression significantly inhibited the luciferase activity of the LINC00511-WT group in BGC823 cells (Fig. 4d). LINC00511 knockdown also significantly upregulated miR-515-5p in BGC823 cells (Fig. 4e). The RIP assay results indicated that both LINC00511 and miR-515-5p were enriched in the mixture immunoprecipitated by anti-Ago2 (Fig. 4f). Moreover, the expression of LINC00511 negatively correlated with miR-515-5p in gastric cancer tissues (Fig. 4g). Therefore, LINC00511 might interact with miR-515-5p in gastric cancer progression.

### LINC00511 knockdown inhibited tumor growth in vivo

**Fig. 5:**
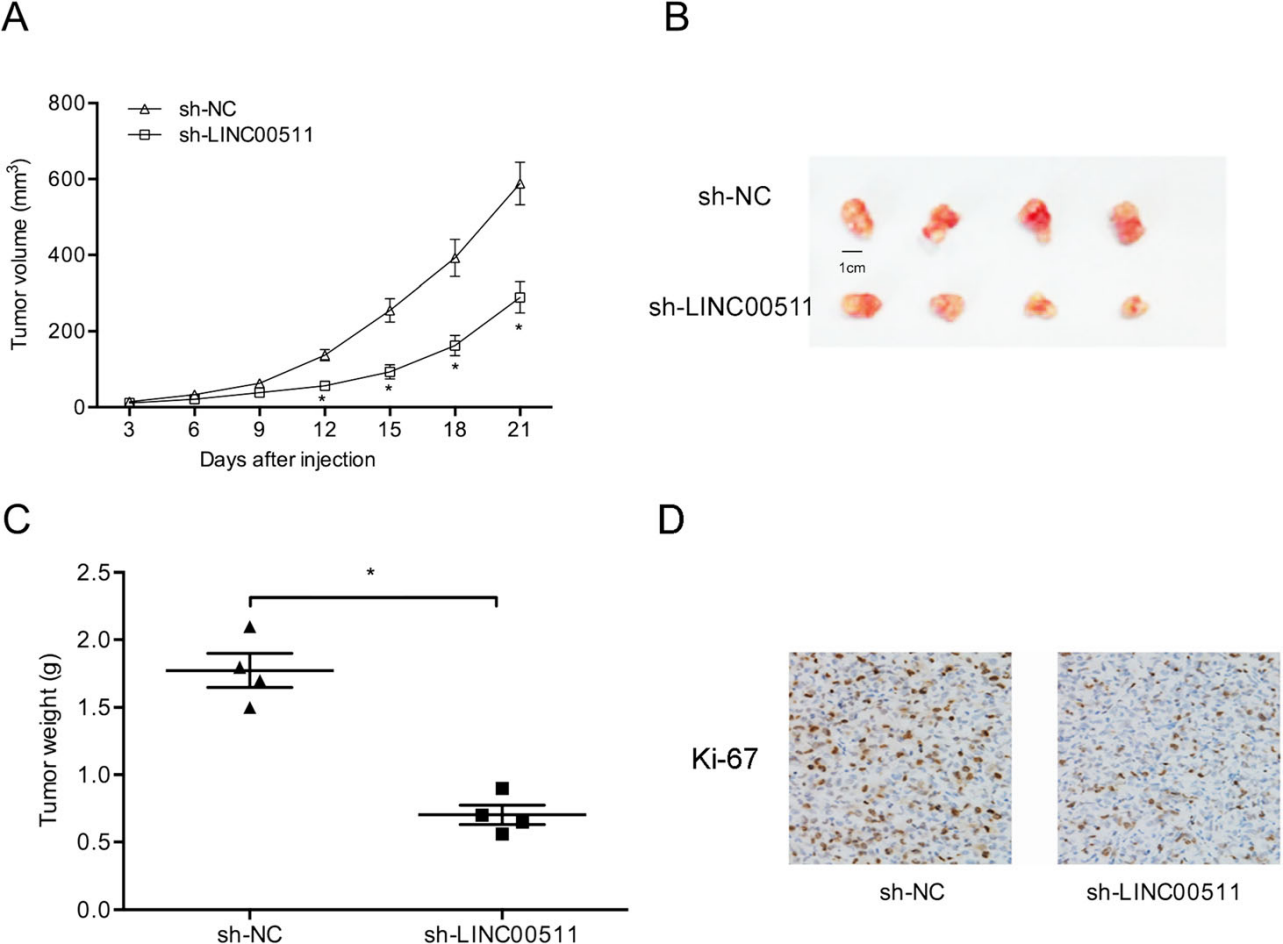
Downregulation of LINC00511 reduces tumor growth. **a**-**c** through Tumor volume growth curves and weights for the sh-LINC00511 group showed inhibition compared to the sh-NC group. **d** Ki67 expression was reduced in sh-LINC00511-treated nude mice. *p < 0.05

We further explored the roles of LINC00511 in tumor growth in vivo. Knockdown of LINC00511 significantly reduced growth of the xenograft of gastric cancer cells (Fig. 5a). Xenograft tumor weights and volumes were also significantly reduced by LINC00511 knockdown (Fig. 5b and c). Furthermore, immunohistochemistry results showed that Ki-67 levels were significantly lower in the sh-LINC0051 group than in the sh-NC group (Fig. 5d). These data revealed that LINC00511 knockdown reduced gastric cancer tumorigenesis in vivo.

## Discussion

Several lncRNAs have recently been identified as having functions in the development and progression of cancers. For example, Liang et al. showed that PTAR promoted ovarian cancer progression by regulating the miR-101-3p/ZEB1 axis [16]. Yang et al. found that HOXA11-AS promoted renal cancer cell proliferation and invasion by targeting the miR-146b-5p/MMP16 axis [17]. Gao et al. found that the MAGI1-IT1/miR-200a/ZEB axis could promote ovarian cancer cell metastasis [18]. However, the roles and underlying mechanisms of lncRNA in tumor progression remain unclear.

LINC00511 (2.265 kb) is a newly discovered lncRNA that is located on chromosome 17q24.3. It is reported to have critical functions in tumorigenesis. For example, Zhao et al. demonstrated that LINC00511 may be a ceRNA that controls VEGFA expression by sponging miR-29b-3p in pancreatic ductal adenocarcinoma [11]. Sun et al. found that LINC00511 promotes lung cancer progression by binding to EZH2 and inhibiting the expression of p57 [19]. Lu et al. showed that the LINC00511/miR-185-3p/E2F1/Nanog axis might play critical roles in breast cancer tumorigenesis and maintenance of stemness [20]. Its function in gastric cancer remains unclear.

We found that LINC00511 was highly expressed in gastric cancer and that this expression positively correlated with larger tumor size, advanced TNM stage and poor disease-free survival. In function assays, LINC00511 knockdown inhibited proliferation, migration and invasion, and reduced tumor growth in vivo. LINC00511 knockdown significantly increased apoptosis and arrested the cell cycle in G0/G1 phase in BGC823 cells. These findings suggest that LINC00511 might act as an oncogenic lncRNA in gastric cancer tumorigenesis.

MAPK signaling pathways have a function in numerous cellular activities, including differentiation, proliferation, metastasis and drug resistance [21, 22]. For example, Song et al. found that the lncRNA ENST00000539653 promoted papillary thyroid cancer progression by regulating one of the MAPK signaling pathways [23]. Yang et al. reported that the lncRNA HOXD-AS1 suppressed proliferation and invasion in colorectal carcinoma by inhibiting the activation of integrin β3 and the MAPK/AKT axis [24]. Liu et al. showed that the NEAT1/let-7a-5p/ Rsf-1 axis regulated nasopharyngeal carcinoma cell cisplatin resistance by regulating the activation of the Ras-MAPK pathway [25]. In our study, the expressions of p-p38, p-ERK and p-JNK were reduced by LINC00511 knockdown in BGC823 cells, suggesting the MAPK signaling pathway promotes the effect of LINC00511 on gastric cancer progression.

Studies have revealed that lncRNAs may act as sponges of miRNAs, further arresting the expression of mRNA [26]. Chen et al. demonstrated that the TTN-AS1/miR-573/E2F3 axis plays critical roles in cervical cancer cell growth and metastasis [27]. Gao et al. revealed that suppressing LUCAT1 reduced glioma cell viability and invasion by modulating miR-375 expression [28]. Previous studies showed that miR-515-5p has critical roles in the progression of many tumors, including prostate cancer [29], lung cancer [30] and breast cancer [12]. However, the function of miR-515-5p in gastric cancer progression remains unclear. In this study, a DIANA analysis showed that LINC00511 possesses a putative miR-515-5p-binding site with a high score. LINC00511 knockdown increased the levels of miR-515-5p in BGC823 cells. Subsequently, overexpression of miR-515-5p significantly downregulated the luciferase activity of the LINC00511-Wt group. The correlation between LINC00511 and miR-515-5p was further confirmed using the RIP assay. In addition, miR-515-5p levels were significantly downregulated and negatively associated with the expression of LINC00511 in gastric cancer tissues. Therefore, we believe that LINC00511 encourages gastric cancer cell proliferation and invasion by sponging miR-515-5p.

## Conclusion

We found that knockdown of LINC00511 could inhibit gastric cancer tumorigenesis through its sponging action on miR-515-5p. Our findings indicate that LINC00511 may be a novel therapeutic target for gastric cancer treatment.

## Data Availability

The datasets supporting the conclusions of this article are included within the article.

## Abbreviations

GC: Gastric cancer
LncRNA: Long non-coding RNAs
miRNAs: MicroRNAs
Mut: Mutant
UTR: Untranslated region
WT: Wild type
